# Defects Identification and Effects of Annealing on Lu_2(1-x)_Y_2x_SiO_5_ (LYSO) Single Crystals for Scintillation Application

**DOI:** 10.3390/ma4071224

**Published:** 2011-07-01

**Authors:** Samuel Blahuta, Aurélie Bessière, Bruno Viana, Vladimir Ouspenski, Eric Mattmann, Julien Lejay, Didier Gourier

**Affiliations:** 1 Ecole Nationale Supérieure de Chimie de Paris (Chimie ParisTech) et Université Pierre et Marie Curie, Laboratoire de Chimie de la Matière Condensée de Paris, UMR–CNRS 7574, 11 rue Pierre et Marie Curie, 75231 Paris Cedex 05, France; E-Mails: aurelie-bessiere@chimie-paristech.fr (A.B.); bruno-viana@chimie-paristech.fr (B.V.); julien-lejay@chimie-paristech.fr (J.L.); didier-gourier@chimie-paristech.fr (D.G.); 2 Saint-Gobain Cristaux et Détecteurs, 104 Route de Larchant, 77140 St-Pierre-lès-Nemours, France; E-Mails: Vladimir.VO.Ouspenski@saint-gobain.com (V.O.); eric.mattmann@saint-gobain.com (E.M.)

**Keywords:** scintillator, electronic defects, thermoluminescence, annealing treatments

## Abstract

The nature, properties and relative concentrations of electronic defects were investigated by Thermoluminescence (TL) in Lu_2(1-x)_Y_2x_SiO_5_ (LYSO) single crystals. Ce and Tb-doped single crystals, grown by the Czochralski technique (CZ), revealed similar traps in TL. LYSO:Ce single crystals were grown by the Floating-Zone technique (FZ) with increasing oxygen concentration in the growth atmosphere. TL intensity is strongly dependent on the oxygen content of the material, and oxygen vacancies are proven to be the main electronic defects in LYSO. The effects of oxidizing and reducing annealing post-treatment on these defects were investigated. While oxidizing treatments efficiently reduce the amount of electronic defects, reducing treatments increase the amount of existing traps. In a thermally assisted tunneling mechanism, the localization of oxygen vacancies around the dopant is discussed. They are shown to be in the close vicinity of the dopant, though not in first neighbor positions.

## 1. Introduction

Ce-doped oxyorthosilicates are efficient scintillating materials used in various fields. Amongst them Lu_2_SiO_5_:Ce (LSO:Ce) was found to be very promising, especially for medical imaging applications such as Positron Emission Tomography (PET) [1,2]. Such interest is due to high stopping power resulting from its high density (7.4 g/cm^3^), high light output (over 30,000 photons/MeV) and fast response (38 ns) [3]. LYSO:Ce was also proposed as an interesting alternative to LSO:Ce [4,5,6]. Introducing Y^3+^ into the matrix reduces the bath temperature during the Czochralski technique (CZ) growth by ~50 °C. Yttrium incorporation has also a positive impact on the melting bath viscosity leading to easier crystal growth and a reduced amount of defects (vacancies, cracks), and is thought to enhance hole mobility in the material. LYSO:Ce shows similar scintillation properties to LSO:Ce and is particularly suitable for PET application due to very good time resolution [7]. Additionally, the luminescence spectra are composed of the electric-dipole allowed 5*d*_1_-4*f* transitions of Ce^3+^ which fit the sensitivity curve of photomultiplier tubes. Since LYSO:Ce is an important material for imaging applications, the detailed study of its scintillation properties is necessary to achieve a better understanding of the scintillation mechanisms [8,9]. As point defects may have a negative impact on scintillation properties (light yield, afterglow, decay time, *etc*.), electronic defects are investigated in this work. The first part of this work focuses on defect identification by using TL. Oxygen vacancies are shown to be the main electronic defects and the trapping parameters are calculated for LYSO:Ce and LYSO:Tb single crystals. The second part of this work deals with annealing treatments as a way to control point defects concentration. The effect of both oxidizing and reducing treatments on TL is presented.

## 2. Results and Discussion

### 2.1. Nature of Electronic Defects

The CZ technique allows growing large single crystals pieces for industrial applications but is very constraining in terms of growth atmosphere because of oxidation of the Ir crucible. As the Floating-Zone technique (FZ) does not require any crucible, it is better suited for testing different atmospheres even though only small crystals can be obtained.

**Figure 1:**
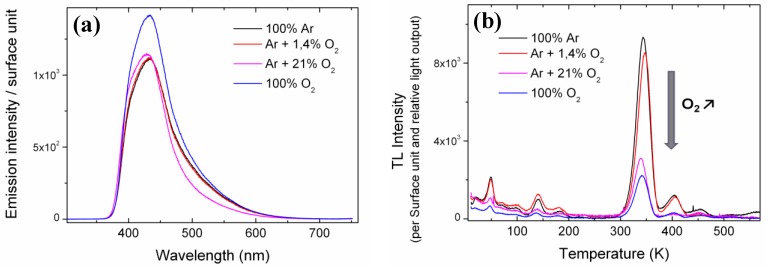
(**a**) Emission spectra under X-Ray excitation at room temperature (RT) of the Floating-Zone technique (FZ) grown samples. The relative light output (LO) calculated by integrating the emission spectra on the 300–700 nm range are 0.82, 0.82, 0.77 and 1 for 100% Ar, Ar + 1.4% O_2_, Ar + 21% O_2_ and 100% O_2_ growth atmospheres, respectively. (**b**) Thermoluminescence (TL) glow curves of the four LYSO:Ce single crystals grown with different oxygen containing atmospheres. All curves are normalized to sample surface and relative LO for comparison. The arrow refers to increasing oxygen content in the growth atmosphere.

For each LYSO:Ce sample grown by the FZ technique, XRL emission spectra were first collected at room temperature (RT) and are shown in Figure 1(a), where the emission amplitude is given per surface unit. The four samples show different luminescence intensity depending on their growth atmosphere. Crystals grown with no oxygen, 1.4% oxygen, or 21% oxygen show lower luminescence than the 100% O_2_ grown crystal. Their relative light output (LO) was calculated by integrating the emission spectra over the 300–700 nm range and are 0.82, 0.82, 0.77 and 1, respectively. The 21% O_2_ sample has a lower relative LO due to differences in its emission spectrum, particularly a weaker contribution around 500 nm. Emission in this wavelength range is ascribed to Ce^3+^ emission in the second crystallographic site (Ce_2_) of LYSO [10]. No explanation has yet been found to explain this difference. 

TL was then performed on the four LYSO:Ce single crystals. The corresponding glow curves are given in Figure 1(b). TL intensities correspond to the integrated emission spectra over the 300–700 nm range. In order to compare the glow curves of all samples, TL intensities were normalized to the sample surface and relative LO previously calculated. For all glow curves, at least five TL peaks can be detected below 250 K. The most intense ones are peaking at 49, 140 and 182 K. At temperatures above 250 K four peaks are present at 347, 404, 454 and 509 K. A fifth peak at 567 K is only found for the crystals grown with 100% Ar, and its relation to oxygen content will be discussed in the second part of this work. All four samples show TL peaks at the same temperatures but with different intensity. Two groups can be distinguished: samples with important TL intensity and samples with about three times less TL. As all glow curves were previously normalized to their relative LO, the TL decrease cannot be ascribed to luminescence quenching. The crystals grown with the highest oxygen content *i.e.*, 21% and 100% oxygen are the ones with the lowest TL intensity whereas crystals grown with 1.4% or no oxygen show higher TL signal. Furthermore, there is a monotonous decrease of the TL intensity with increasing oxygen content in the growth atmosphere. This shows that trapping defects in LYSO that are involved in TL are directly linked to the oxygen content in the crystal. More precisely they are related to an oxygen deficiency in the material: the less oxygen in the growth atmosphere, the more intense the TL. These results bring a first substantial proof of the nature of the electronic defects responsible for the main TL peaks in LYSO:Ce *i.e.*, oxygen vacancies as it had been suggested in earlier works [11,12]. 

**Figure 2:**
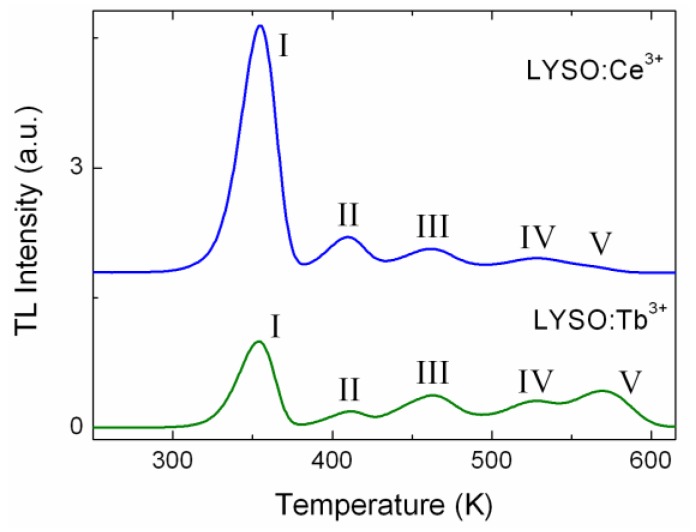
TL glow curves of LYSO:Ce and LYSO:Tb single crystals. Both samples are 10 × 10 × 1 mm^3^ with the largest faces polished. Heating rate was set to 20 K/min.

The comparison of the nature of the defects in LYSO:Ce and LYSO:Tb was also investigated by TL. LYSO:Tb shows low light yield (12,000 Photons/MeV) compared to LYSO:Ce (30,000 Photons/MeV). Figure 2 shows their respective glow curves from 250 K to 620 K. Both samples show five peaks at similar temperatures (±3 K): 354, 410, 462, 524 and 569 K. For Ce-doped LYSO the last peak is very weak, thus the temperature value is given for LYSO:Tb. The comparison with the TL glow curves of the FZ grown samples reveals they all show the same peaks above 250 K, except for the last peak which is not always present. Small differences in temperature for each peak are ascribed to thermal lag due to the set-up. The TL peaks above 250 K will be identified as peak I to peak V in the order of increasing peak temperature. Figure 2 shows that for Ce and Tb-doped LYSO, the TL peaks above 250 K are at similar temperatures but show different relative intensities. Hence this result suggests that the nature of the corresponding traps does not depend on the doping ion, the latter may just influence their relative concentrations. This confirms the identification of oxygen vacancies as responsible for the main TL peaks.

**Figure 3:**
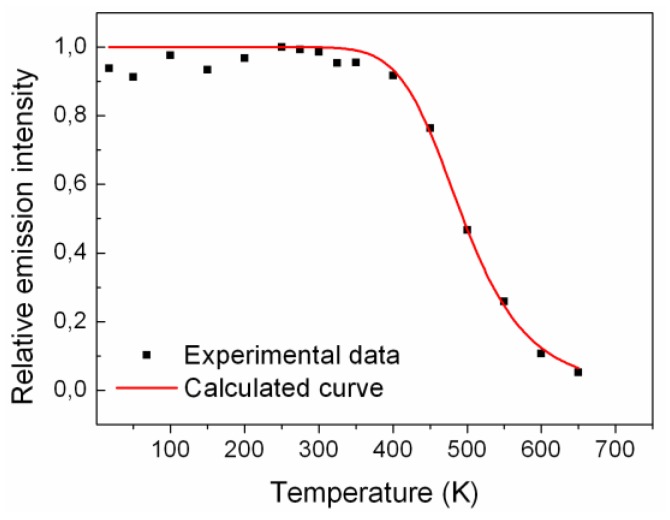
Temperature dependence of the light output for LYSO:Ce determined from integrated emission spectra. The curve is the fit to Equation (1).

The characteristic properties of each trap were calculated for both LYSO:Ce and LYSO:Tb. For comparison, TL glow curves need to be corrected for their respective thermal quenching. In LYSO:Ce the emission spectra correspond to Ce^3+^ 5*d_1_*-4*f* transitions. It was shown by Feng *et al.* [13] that thermally induced ionization of the Ce^3+^ 5*d_1_* state can be followed by a delayed radiative recombination of the escaped electron with Ce^4+^ centers. Thus, ionization of the 5*d_1_* state does not necessarily lead to luminescence quenching. By taking this into account, it is necessary to figure out whether the delayed recombination process has to be considered to correct TL glow curves. As the TL measurements were performed with a 2 s aperture, we have to consider this delayed process and take into account the whole emission, also called steady-state emission by Feng *et al.* The evolution of the emission intensity with temperature was measured from 650 K to 10 K under X-ray excitation and is shown in Figure 3. For each temperature (every 50 K) the emission spectrum was integrated over the 300–700 nm range. The data were then fitted using the Mott-Gurney formula (Equation (1)):

(1) $$Q\left(T\right)=\frac{1}{1+\frac{k_{n}}{k_{r}}exp\left(-\frac{E_{QT}}{kT}\right)}$$

where k_r_ and k_n_ are the probabilities or radiative and nonradiative transitions from the Ce^3+^ excited state to the ground state, respectively, and k = Boltzmann’s constant = 8.617 × 10^−5^ eV/K. As these measurements take both the prompt and delayed radiative recombination processes into account, the numerical interpolation given by Equation 1 (see Figure 3) can be used to correct the TL glow curve. The calculated parameters were: E_QT_ = 0.47 ± 0.05 eV, and $k_{n}/k_{r}$: 69,958 ± 8,301. As the delayed luminescence process is included, the calculated values for E_QT_ and $k_{n}/k_{r}$ cannot be compared to that published in other works [14].

For Tb doped LYSO, the emission is only composed of the Tb^3+^ 4*f*-4*f* lines with maximum emission at 545 nm. As 4*f* electrons are quite insensitive to crystal field variations, and lattice vibrations (weak electron-phonon coupling), 4*f*-4*f* transitions usually show no thermal extinction. Hence LYSO:Tb glow curve was not corrected.

**Figure 4:**
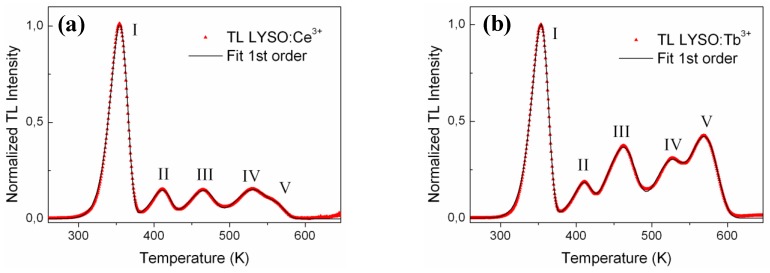
Corrected TL glow curves (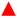) and fitting curves (—) of (**a**) LYSO:Ce and (**b**) LYSO:Tb samples. Each curve is fitted with five 1st order kinetic peaks.

TL glow curves were then fitted using Kitis *et al.* equations given in refs [15,16]. Each peak was first fitted with both 1st and 2nd order kinetic equations to determine which one was most suitable. It was found that the five TL peaks show first order kinetic as described by Equation (2). This was also shown in a previous work by Vedda *et al.* [12] but only for the peaks I to IV, peak V being ascribed to 2nd order kinetic and only present for LYSO but not for LSO [12]. The whole glow curve was then fitted by the sum of five 1st order kinetic peaks, namely peaks I, II, III, IV and V. The values for the temperature at peak maximum: T_m,i_ and the intensity I_i_ with I = 1 to 5 were taken from the experimental data. Each glow curve was fitted with five parameters corresponding to the energy or trap depth: E_i_, i = 1 to 5. Figure 4(a) and Figure 4(b) show the corrected glow curves and the fitting curves for LYSO:Ce and LYSO:Tb, respectively. The iterative Levenberg-Marquardt fitting algorithm was applied in the 250–620 K range and yielded to a R² value greater than 0.999. The most relevant parameters for the corresponding traps (*i.e.*, E and T_m_) are given in Table 1. The frequency factor *s* was calculated using Equation (3) and is also given in Table 1 for each trap.

(2) $$I\left(T\right)=I_{m}\;exp\left[1+\frac{E}{kT}\frac{T-T_{m}}{T_{m}}-\frac{T^{2}}{T_{m}^{2}}exp\;\left\{\frac{E}{kT}\frac{T-T_{m}}{T_{m}}\right\}\left(1-\Delta \right)-\Delta_{m}\right]$$

with Δ=2kT/E and $\Delta_{m}=2kT_{m}/E$

(3) $$\frac{\beta E}{kT_{m}^{2}}=s\;exp\left\{-\frac{E}{kT_{m}}\right\}$$

where β is the heating rate = 0.33 K/s.

**Table 1:** Trap parameters E, T_m_ and s for the fitted glow curves of LYSO:Ce and LYSO:Tb. All peaks are characteristic of first order kinetic.

	***LYSO:Ce***			***LYSO:Tb***		
TL peak	E (eV)	T_m_ (K)	s (s^−1^)	E (eV)	T_m_ (K)	s (s^−1^)
I	0.941 ± 0.002	354	7.0 × 10^11^ ± 2.9 × 10^9^	0.963 ± 0.002	353	1.5 × 10^12^ ± 6.1 × 10^9^
II	1.14 ± 0.02	410	2.7 × 10^12^ ± 9.1 × 10^10^	1.21 ± 0.02	408	2.5 × 10^13^ ± 7.8 × 10^11^
III	1.12 ± 0.02	464	2.9 × 10^10^ ± 1.0 × 10^9^	0.981 ± 0.006	461	9.2 × 10^8^ ± 1.1 × 10^7^
IV	1.24 ± 0.04	526	1.3 × 10^10^ ± 9.2 × 10^8^	1.28 ± 0.02	520	4.7 × 10^10^ ± 1.3 × 10^9^
V	1.70 ± 0.41	558	4.8 × 10^13^ ± 3.1 × 10^13^	1.38 ± 0.01	569	2.8 × 10^10^ ± 5.3 × 10^8^

All calculated traps depths are found to be around 1–2 eV which is consistent with the literature [12,17]. For Ce-doped LYSO calculated errors are less than 0.04 eV for peaks I to IV and 0.4 eV for peak V due to weaker intensity. For the Tb-doped sample all energy values have an error of less than 0.02 eV. According to the values given in Table 1 the trap depths in LYSO:Ce and LYSO:Tb only differ from less than 0.15 eV (peak V is not taken into account due to the lack of precision). Hence we assume that both compositions have similar trap depth. Larger differences are observed for the frequency factor *s*. This parameter is characteristic of the trap-to-emitting center transfer efficiency. As proposed by Vedda *et al.* we assume a thermally activated tunneling transfer from the traps in an excited state to the luminescent centers [12]. As *s* depends exponentially on the distance *r* between the trap and the center, and as Ce^3+^ is 8% larger than Tb^3+^, the trap-to-center distances are not expected to be the same in both materials. Thus *s* may vary with the dopant by up to one order of magnitude when *r* increases by 10%. These calculations confirm our previous assessment: electron traps in LYSO corresponding to peaks above 250 K just depend on the matrix and not on the dopant. Hence, traps located above 250 K are clearly ascribed to oxygen vacancies. Unlike the results previously published by Vedda *et al.* [12], the calculated trap depths are not all equal. This difference can be explained by the different environment of all the oxygens, especially for LYSO where two possible neighboring cations are possible, *i.e*., Lu and Y. Concerning TL peaks below 250 K, their dependence on the oxygen content during the growth (Figure 1(b)) also suggests that they are related to oxygen vacancies. Nevertheless, the origins of these shallow traps are not yet well established.

**Figure 5:**
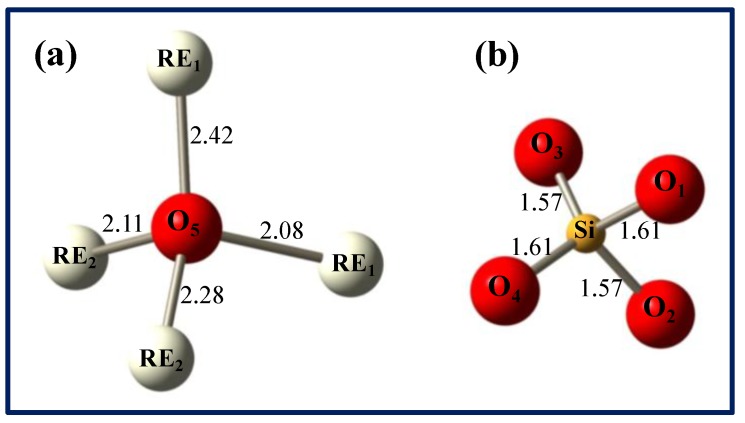
Environment of the five oxygen atoms in the LYSO lattice (distances in Å): non-silicon bound O_5_ (**a**) and SiO_4_ tetrahedron (**b**). RE stands for Lu^3+^/Y^3+^ atoms and distances are a mean value of Lu-O and Y-O distances.

The formation of oxygen vacancies can be understood from the crystalline structure of the LYSO host. It is made of SiO_4_ tetrahedra linked to one rare earth (RE = Lu/Y). The RE occupy two different sites, namely RE_1_ and RE_2_ with 7 and 6 neighboring oxygens, respectively. Five different oxygen sites are distinguished: O_1_ to O_4_ are related to SiO_4_ polyhedra whereas O_5_ is not bonded to silicon and is solely linked to Lu/Y, thus forming [O-RE_4_] tetrahedra. Figure 5 shows the environment of the different oxygen atoms with the main bonding distances in LYSO with 10% Y [18]. Oxygen vacancies can be created during CZ growth due to low oxygen content in the furnace atmosphere. It was shown that vacancies of the fifth oxygen (O_5_) have the lowest formation energy and that doubly positive charged vacancies are energetically favorable [19]. Thus, $V_{O_{5}}^{●●}$ vacancies (in Kröger-Vink notation) are the most likely defects, which act as electron traps.

The scintillation mechanism occurring during XRL is first the trapping of a hole by Ce^3+^ forming temporary Ce^4+^, followed by the capture of a free electron resulting in Ce^3+^ in an excited state, noted (Ce^3+^)*. The latter de-excites radiatively giving a blue photon characteristic of the 5*d_1_*-4*f* transition. For TL, during the sample irradiation at low temperature, holes are trapped at Ce centers and electrons are trapped at oxygen vacancies. When the irradiation is stopped and the sample is being heated electrons progressively de-trap and recombine at Ce centers, leading to Ce^3+^ 5*d_1_*-4*f* emission. 

The localization of the oxygen vacancies is an interesting question to understand the TL mechanism, since it was shown to be a thermally assisted tunneling process by Vedda *et al.* [12]. We propose that they are mainly created in the proximity to the doping RE ions. A first indication that vacancies are localized near the dopants comes from the fact that both Ce^3+^ and Tb^3+^ replace cations of smaller size (*i.e.*, Lu^3+^ and Y^3+^). A deformation of the lattice is thus expected and could be absorbed on shorter distances by the creation of vacancies around the dopant. Secondly, Clabau *et al.* claim that anion vacancies tend to settle in the vicinity of the dopant ions due to efficient delocalization of their electronic density on the surroundings [20]. This is explained by the lower ionization potential of Ce^3+^ with respect to that of host cations Lu^3+^ and Y^3+^. Thus Ce^3+^ has a tendency to attract anion vacancies in its surrounding. According to these arguments it seems reasonable to assume that oxygen vacancies are located close to the dopant ions. Nevertheless, vacancies in the very first coordination sphere of Ce^3+^ are not expected. Indeed, Electron Paramagnetic Resonance studies on LSO:Ce by Pidol *et al.* did not show any additional splitting for Ce^3+^ (other than the two sites Ce_1_ and Ce_2_) as would be expected if an oxygen vacancy was located next to the dopant for some Ce^3+^ ions [10]. An oxygen vacancy in the first coordination sphere of Ce^3+^ would lead to a perturbed EPR spectrum due to important changes in the orientation of the g tensor axes. Furthermore, as all O_5_ are not equidistant from the RE sites, all V_O5_ are not magnetically equivalent and additional splitting would be present. Another justification that oxygen vacancies are not located in the first coordination sphere of Ce^3+^ is the unchanged luminescence characteristics. Otherwise, the local crystal field would be dramatically impacted and the emission would be red-shifted. Consequently, we propose that the introduction of Ce^3+^ in the host lattice is accompanied by oxygen vacancies in its surrounding, but not as first neighbors. As electron tunneling in such materials is not expected to be efficient over distances >10 Å [21], oxygen vacancies evidenced in TL must be located within a few interatomic distances from Ce^3+^.

**Figure 6:**
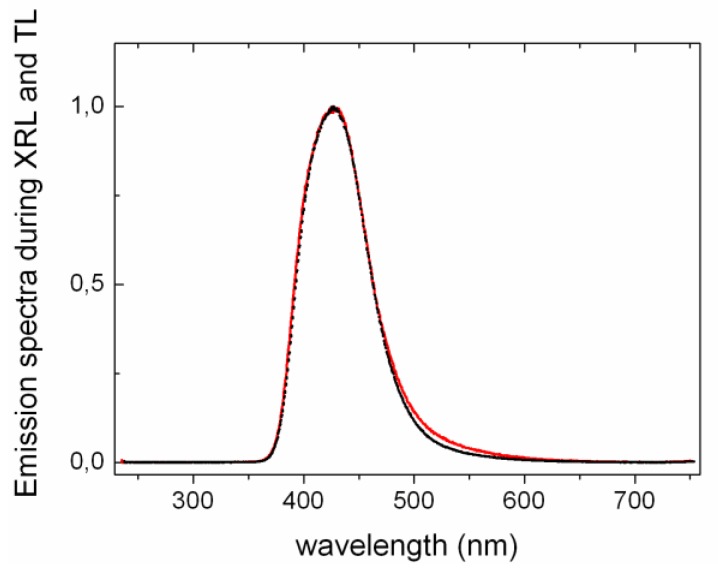
Normalized emission spectra of Ce-doped LYSO during TL (dashed black line) and XRL (continuous red line) measurements. All spectra were measured at 370 K. LYSO:Ce shows Ce^3+^ 5d-4f emission. The resolution is different due to larger slit opening during TL measurement.

Furthermore it is interesting to compare the shape of TL and XRL emission spectra as depicted in Figure 6 for LYSO:Ce. Assuming a thermally activated tunneling recombination as discussed in ref. [12], TL is the consequence of electron detrapping in the neighborhood of the doping ions. On the contrary, XRL involves excitation of the luminescent centers via bandgap excitation and therefore originates from all Ce^3+^ ions and not only those close to electronic traps *i.e.*, oxygen vacancies. Anionic vacancies around the dopant are expected to influence the local crystal field and therefore to affect the energy of the 5*d*-4*f* transitions. Nevertheless, as Ce^3+^ 5*d*-4*f* transitions have a strong electron-phonon coupling, the small induced crystal field perturbations will hardly modify the emission spectrum. Consequently, XRL and TL emission spectra appear identical as shown in Figure 6. This is also consistent with the identical emission spectra of LYSO:Ce during photoluminescence and radioluminescence obtained by Feng *et al.* [13].

It is difficult to estimate the amount of electron traps in our materials, even by integrating TL glow curves which underestimates this amount. Oxyorthosilicates with electronic trap density of 10^18^ traps/cm^3^ were shown to be efficient materials for storage devices [22]. For PET application, this density is much lower, and 10^15^–10^17^ traps/cm^3^ seems to be a reasonable approximation [23]. If we only consider O_5_ oxygens, which are believed to be the main sites of oxygen vacancies, the LYSO lattice contains 10^22^ oxygen/cm^3^. Thus considering 10^15^–10^17^ oxygen vacancies per cm^3^ leads to one O_5_ vacancy out of 10^7^–10^5^ O_5_, which is a reasonable assessment for vacancy creation. Furthermore, by taking the example of Ce-doped LYSO with initial concentration of 0.22 at%, Glow Discharge Mass Spectrometry gives a final amount of 250 wt/ppm Ce in the crystal which corresponds to 10^18^ Ce/cm^3^. For each Ce with neighboring oxygen vacancies, the ones which participate to TL, we define a so-called close vicinity which contains the vacancies and which is in a radii of about 10 Å around the dopant.

The first part of this work gave strong arguments that oxygen vacancies are the main electronic defects in LYSO single crystals. These vacancies act as electron traps and may play different roles depending on their depth. Shallow traps (represented by TL peaks below RT) should have a negative impact on response time as they create intermediate steps for the charge carriers [24]. Deeper traps (TL peaks above RT) are responsible for the important afterglow in LYSO:Ce but may also impact the light yield during the application. Indeed, thermal energy required to empty high temperature traps will not be reached at RT [25]. Thus it is of primary interest to find efficient ways to reduce the amount of traps in LYSO, *i.e.*, to reduce the number of oxygen vacancies [11,26]. From an application point of view, CZ growth is the most efficient way to produce large LYSO pieces. FZ technique was used as a tool to experiment different growth atmospheres. The following results have been obtained on LYSO:Ce single crystals grown by CZ. 

### 2.2. Effects of Annealing Treatments

**Figure 7:**
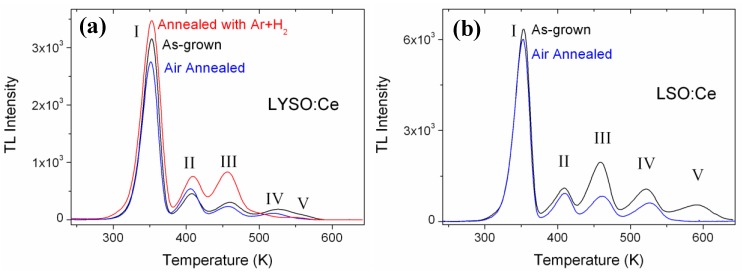
TL glow curves of: (**a**) LYSO:Ce single crystals as-grown, after reducing annealing and after air annealing; (**b**) LSO:Ce as-grown and after air annealing. For comparison, all TL intensities are normalized to the sample surface and to their respective light output.

As oxygen vacancies were identified to be the main electron traps in oxyorthosilicates, the effect of annealing treatment was investigated. One 10 × 10 × 1 mm LYSO:Ce single crystal was annealed in air at 1,500 °C for 48 hours and characterized by TL. The comparison of its TL glow curve with that of an as-grown sample is shown in Figure 7(a). Another crystal with the same geometry was annealed with Ar + 5% H_2_ at 1,200 °C for 12 hours. The glow curves are not corrected for thermal quenching but all TL intensities are normalized to the sample surface and relative LO. For these three samples, the respective LO were measured to be 1 (as-grown crystal), 1.16 (after air annealing) and 0.85 (after reducing annealing). 

We found an improvement of the LO after air annealing in line with the increase already reported for LYSO:Ce by Chai *et al.* [27]. Here the decrease of the LO with the reducing treatment is explained by the increase of oxygen vacancies that are either quenching centers or deep traps centers. TL measurements reveal that the sample annealed in a reducing atmosphere shows more TL intensity, except for peaks IV and V. More oxygen vacancies are therefore created with an additional treatment in reducing conditions. On the contrary, the air annealed sample shows less TL intensity, except for peak II at 410 K. However, the reduction of the TL intensity following air annealing is rather weak. As a comparison, larger annealing effect is observed for peaks II to V in LSO:Ce (shown in Figure 7(b)). It seems that the reduction of oxygen vacancies is more efficient in LSO:Ce than in LYSO:Ce sample. The decrease of TL in LSO:Ce is in agreement with the data reported by Ding *et al.* [28], where a TL decrease of about 40% was observed for a LSO:Ce crystal annealed in air at 1,400 °C for 10 h. The greater decrease of TL in LSO:Ce compared to LYSO:Ce may be explained by a more important initial defect concentration as peak II to V were more important relatively to peak I in the LSO sample. Hence the improvement that can be obtained by an air annealing treatment is greater when defects are initially more important, whatever the nature of the host (LSO or LYSO). 

One particular point is important to notice concerning peak V in Figure 7(b). Although the corresponding traps are emptied at the highest temperature (580–600 K), they are the first defects to disappear following air annealing. We have also observed that air annealing at lower temperature (1,200 °C) is sufficient to suppress peak V without impacting any other peak. The analogy can also be made with FZ grown samples where peak V only appears when no oxygen is present in the growth atmosphere (100% Ar). This can be explained as follows. From a kinetic point of view, traps corresponding to peak V require the highest energy to be emptied (TL), but from a thermodynamic point of view they are the less stable as they disappear easily with annealing. Dorenbos *et al.* [17] and Vedda *et al.* [12] proposed a thermally activated tunneling trap-to-center mechanism for TL in LSO:Ce. The defects being of same nature (*i.e.*, oxygen vacancies), each trap has the same energy depth, but differs from the others by its frequency factor that is related to the trap-to-luminescent center distance as discussed above. Consequently the highest temperature peaks correspond to the oxygen vacancies at the longer distances from the Ce^3+^ center. Hence we found that vacancies close to Ce^3+^ (lower temperature TL peaks) will hardly diffuse out of the lattice during annealing due to important constraints and stabilization by Ce^3+^ electronic delocalization. On the contrary, vacancies at longer distances from Ce^3+^ (higher temperature TL peaks) will be the first ones to disappear during oxidizing annealing. Peak V is the perfect example, but we have also observed this effect for the other peaks. In a recent work [12] it was proposed that peak V is only visible in LYSO, while LSO only displays four TL peaks. Here we show that this is not the case. The presence of a peak V in orthosilicates (LYSO, LSO, YSO) is rather correlated to the history of the crystal. Peak V is missing if the crystal is grown with enough oxygen, as for FZ grown samples with 1.4%, 21% and 100% oxygen. For CZ grown samples where very low oxygen content is required, oxidizing post-treatment allows filling oxygen vacancies corresponding to peak V. TL is thus also an efficient tool to determine whether or not a crystal has been annealed in an oxidizing atmosphere.

## 3. Experimental Section

### 3.1. Crystal Growth

**Figure 8:**
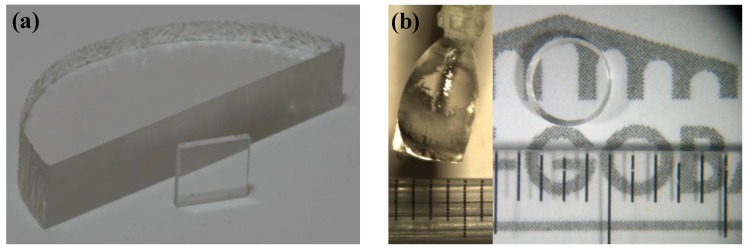
(**a**) LYSO:Ce single crystal grown by Czochralski technique (CZ); (**b**) LYSO:Ce single crystals grown by the floating zone technique (FZ) with 100% Argon atmosphere (left: as-grown and right: after cutting and polishing).

The LYSO:Ce and LYSO:Tb single crystals with dopant nominal content of 0.11 at% were grown by the CZ technique and were provided by Saint-Gobain Crystals, France. The nominal compositions in the bath correspond to Lu_1.798_Y_0.1998_ Ce_0.0022_ SiO_5_ and Lu_1.798_Y_0.1998_ Tb_0.0022_ SiO_5_, with Y content being 10 at% of the rare earths. High purity Lu_2_O_3_, Y_2_O_3_, SiO_2_, CeO_2_ and Tb_2_O_3_ were used as starting powders. The mix of powders was heated around 2,050 °C in an iridium (Ir) crucible. The latter being very sensitive to oxygen, the growth was performed under a nitrogen flux with very low oxygen content. Samples with size of 10 × 10 × 1 mm^3^ were polished on both large faces before experiments (Figure 8(a)). Post growth annealing treatments were then performed either with air for oxidizing conditions or with Argon + 5% H_2_ for reducing conditions. In addition, CZ grown LSO:Ce single crystals from previous studies were also characterized for comparison [10].

In order to allow growth atmosphere with larger oxygen content than during CZ, other LYSO:Ce single crystals were grown by the Optical Floating-Zone (FZ) technique. Lu_2_O_3_, Y_2_O_3_, CeO_2_ and SiO_2_ starting powders were mixed and ground thoroughly. The nominal composition was Lu_1.798_ Y_0.1998_ Ce_0.0022_ SiO_5_. This powder mixture was shaped into four, 3 mm-diameter and 100 mm-long cylindrical bars under an isostatic pressure of 700 kg/cm^2^. These bars were then sintered in air at 1,500 ^o^C for 13 hours, ground once more into powder, reshaped into bars and sintered a second time under the same conditions. This two-step preparation ensures a complete phase formation, confirmed by X-ray diffraction (not shown) which revealed pure monoclinic C2/c phase, and an increased density of the bars. These bars were then placed in a Cyberstar mirror furnace apparatus (1,000 W halogen lamp) in a quartz tube with controlled flowing gas atmosphere. A single crystal of the same composition was used as a seed. The controlled atmosphere was 100% oxygen, 21% oxygen in argon, 1.4% oxygen in argon or 100% argon (the % values are by volume). Four transparent and colorless crystals were obtained. Despite the presence of cracks, 1mm-thick single crystalline samples with surface of around 20 mm^2^ were cut and polished (Figure 8(b)).

### 3.2. Characterization

TL measurements were performed on 1 mm thick polished single crystals. The samples were glued with silver paint to a copper sample holder attached to the cold head of a Janis cryostat and placed under vacuum. The sample was first excited in situ at 10 K for 10 minutes by a molybdenum X-ray source operating at 50 kV and 20 mA. The beam goes through a Beryllium window in the cryostat and excites the sample surface at a 45° angle. The sample was heated at 20 K/min between 10 K and 650 K using a LakeShore 340 temperature controller.

Luminescence was collected via an optical fiber by a Princeton CCD camera cooled to −65 °C coupled with an Acton SpectraPro 1250i monochromator equipped with a grating to allow spectral resolution. The emitted light was collected from the irradiated side of the sample at 45° to the sample surface.

The same detection equipment was used for measuring radioluminescence under X-ray excitation (XRL). The CCD camera shutter aperture was set to 1 s for XRL and 2 s for TL, allowing good signal to noise ratio. 

## 4. Conclusions

LYSO single crystals were prepared in order to investigate both the nature and the concentration of electronic defects. Ce-doped LYSO grown with different oxygen containing atmospheres has allowed highlighting the dependence of TL intensity on the oxygen content of the lattice. Further investigations on LYSO:Tb confirmed that the electronic defects are not dopant-dependent. 

Both data evidence that defects responsible for TL in LYSO single crystals are due to oxygen vacancies that behave as electron traps in the material. According to the TL parameters, oxygen vacancies are expected to be in a close neighborhood of the doping ion, but without being in its first coordination sphere. To ensure efficient trap-to-centre recombination by tunneling, a so-called close vicinity which contains the oxygen vacancies is defined within 10 Å around the dopant and is estimated to involve up to one Ce out of ten. 

The impact of annealing treatments on electronic defects in LYSO:Ce single crystals was investigated. Air annealing at 1,500 °C was shown to be an efficient way to reduce TL intensity (*i.e.*, the amount of oxygen vacancies) while annealing in a reducing atmosphere has the opposite consequence. Peak V (around 580 K), present in LYSO:Ce as well as in LSO:Ce, can be easily removed by annealing in air. It corresponds to the furthest oxygen vacancy from Ce^3+^ and as such is the less stabilized by the proximity to Ce^3+^.

## References

[1] Melcher C.L. (1991). Lutetium orthosilicate single crystal scintillator detector. U.S. Patent.

[2] Melcher C.L., Schweitzer J.S. (1992). A promising new scintillator: Cerium-doped lutetium oxyorthosilicate. Nucl. Instrum. Meth. A.

[3] Van Eijk C.W.E. (2002). Inorganic scintillators in medical imaging. Phys. Med. Biol..

[4] Cooke D.W., McClellan K.J., Bennett B.L., Roper J.M., Whittaker M.T., Muenchausen R.E., Sze R.C. (2000). Crystal growth and optical characterization of cerium-doped Lu_1.8_Y_0.2_SiO_5_. J. Appl. Phys..

[5] Chai B., Ji Y. (2003). Lutetium yttrium orthosilicate single crystal scintillator detector. U.S. Patent.

[6] McClellan K.J. (2001). Single crystal scintillator. U.S. Patent.

[7] Chewpraditkul W., Swiderski L., Moszynski M., Szczesniak T., Syntfeld-Kazuch A., Wanarak C., Limsuwan P. (2009). Scintillation properties of LuAG:Ce, YAG:Ce and LYSO:Ce crystals for gamma-ray detection. IEEE Trans. Nucl. Sci..

[8] Pauwels D., Le Masson N., Viana B., Kahn-Harari A., Van Loaf E.V.D., Dorenbos P. (2000). A novel inorganic scintillator: Lu_2_Si_2_O_7_ :Ce^3+^ (LPS). IEEE Trans. Nucl. Sci..

[9] Pidol L., Kahn-Harari A., Viana B., Virey E., Ferrand B., Dorenbos P., De Haas J.T.M., Van Eijk C.W.E. (2004). High efficiency of lutetium silicate scintillators, Ce-doped LPS, and LYSO crystals. IEEE Trans. Nucl. Sci..

[10] Pidol L., Guillot-Noël O., Kahn-Harari A., Viana B., Pelenc D., Gourier D. (2006). EPR study of Ce^3+^ ions in lutetium silicate scintillators Lu_2_Si_2_O_7_ and Lu_2_SiO_5_. J. Phys. Chem. Solids.

[11] Pidol L., Viana B., Kahn-Harari A., Ferrand B, Dorenbos P., Van Eijk C.W.E. (2005). Scintillation and thermoluminescence properties of Lu_2_Si_2_O_7_: Ce^3+^ crystals. Nucl. Instrum. Meth. A.

[12] Vedda A., Nikl M., Fasoli M., Mihokova E., Pejchal J., Dusek M., Ren G., Stanek C.R., McClellan K.J., Byler D.D. (2008). Thermally stimulated tunneling in rare-earth-doped oxyorthosilicates. Phys. Rev. B.

[13] Feng H., Jary V., Mihokova E., Pejchal J., Dusek M., Ren G., Stanek C., McClellan K., Byler D. (2010). Temperature dependence of luminescence characteristics of Lu_2(1-x)_Y_2x_SiO_5_:Ce^3+^ scintillator grown by the Czochralski method. Phys. Rev. B.

[14] Yang K., Melcher C.L., Rack P.D., Eriksson L. (2009). Effects of calcium codoping on charge traps in LSO:Ce crystals. IEEE Trans. Nucl. Sci..

[15] Bos A. (2006). Theory of thermoluminescence. Radiat. Meas..

[16] Kitis G., Gomez-Ros J.M., Tuyn J.W.N. (1998). Thermoluminescence glow-curve deconvolution functions for first, second and general orders of kinetics. J. Phys. D.

[17] Dorenbos P., Van Eijk C.W.E., Bos A.J.J., Melcher C.L. (1994). Afterglow and thermoluminescence properties of Lu_2_SiO_5_:Ce scintillation crystals. J. Phys.: Condens. Matter.

[18] Chiriu D., Faedda N., Lehmann A.G., Ricci P.C., Anedda A., Desgreniers S., Fortin E. (2007). Structural characterization of Lu_1.8_Y_0.2_SiO_5_ crystals. Phys. Rev. B.

[19] Liu B., Qi Z., Gu M., Liu X., Huang S., Ni C. (2007). First-principles study of oxygen vacancies in Lu_2_SiO_5_. J. Phys.: Condens. Matter.

[20] Clabau F., Rocquefelte X., Le Mercier T., Deniard P., Jobic S., Whangbo M.H. (2006). Formulation of phosphorescence mechanisms in inorganic solids based on a new model of defect conglomeration. Chem. Mater..

[21] Cohen-Tannoudji C., Diu B., Laloë F. (1997). Mécanique Quantique.

[22] Anneda A., Ricci P., Chiriu D. (2008). Doped rare earths orthosilicates used as optical devices for recording information. WO Patent.

[23] Vedda A. (2011). Personal communication.

[24] Wojtowicz A., Szupryczynski P., Wisniewski D., Glodo J., Drozdowski W. (2001). Electron traps and scintillation mechanism in LuAlO_3_:Ce scintillators. J. Phys.: Condens. Matter.

[25] Bartram R.H., Hamilton D.S., Kappers L.A., Lempicki A. (1997). Electron traps and transfer efficiency of cerium-doped aluminate scintillators. J. Lumin..

[26] Blahuta S., Viana B., Bessière A., Mattmann E., LaCourse B. (2011). Luminescence quenching processes in Gd_2_O_2_S:Pr^3+^,Ce^3+^ scintillating ceramics. Opt. Mater..

[27] Chai B. (2007). Method of enhancing performance of cerium doped lutetium yttrium orthosilicate crystals and crystals produced thereby. U.S. Patent.

[28] Ding D., Feng H., Ren G., Nikl M., Qin L., Pan S., Yang F. (2010). Air atmosphere annealing effects on LSO:Ce crystal. IEEE Trans. Nucl. Sci..

